# Survival prediction modelling in patients with acute ST-segment elevation myocardial infarction with LASSO regression and explainable machine learning

**DOI:** 10.3389/fmed.2025.1594273

**Published:** 2025-07-18

**Authors:** Tao Shi, Jianping Yang, Yanji Zhou, Sirui Yang, Fazhi Yang, Xinuo Ma, Yujuan Peng, Jinfang Pu, Hong Wei, Lixing Chen

**Affiliations:** ^1^Department of Cardiology, The First Affiliated Hospital of Kunming Medical University, Kunming, China; ^2^College of Big Data, Yunnan Agricultural University, Kunming, China; ^3^The Key Laboratory for Crop Production and Smart Agriculture of Yunnan Province, Kunming, China; ^4^Department of Pediatrics, The First Affiliated Hospital of Kunming Medical University, Kunming, China

**Keywords:** acute ST-segment elevation myocardial infarction, LASSO regression, explainable machine learning, Cox proportional hazards regression, random survival forest

## Abstract

**Background:**

Acute ST-segment elevation myocardial infarction (STEMI) is a cardiovascular emergency that is associated with a high risk of death. In this study, we developed explainable machine learning models to predict the overall survival (OS) of STEMI patients to help improve prognosis and increase survival.

**Methods:**

After applying the inclusion and exclusion criteria, we selected 893 patients who underwent emergency coronary angiography and percutaneous coronary intervention (PCI) for STEMI at the First Affiliated Hospital of Kunming Medical University. The best predictor variables were screened by least absolute shrinkage and selection operator (LASSO) regression. These variables were used to construct Cox proportional hazards regression (coxph) and random survival forest (rfsrc) models. Three criteria (C-index, Brier score, and C/D AUC) were utilised to compare the performance of the two models. Then, by applying the time-dependent variable importance and the partial dependence survival profile, a global explanation of the entire cohort was conducted. Finally, local explanations for individual patients were performed with the SurvSHAP(t) and SurvLIME plots and the ceteris paribus survival profile.

**Results:**

Combining the results of the comparison of the three criteria, the performance of the rfsrc model was shown to be superior to that of the coxph model. LASSO regression was used to screen 11 predictor variables, such as diastolic blood pressure (DBP), Killip class, hyperlipidaemia, global registry of acute coronary events (GRACE) Score, creatine kinase isoenzyme-MB, myoglobin, white blood cells, monocytes, thrombin time, globulin (GLB), and conjugated bilirubin. The global explanation of the whole cohort revealed that DBP, GRACE Score, myoglobin, and monocytes had a significant effect on the OS of STEMI patients in the coxph model and that DBP, GRACE Score, and GLB were the variables that significantly affected the OS of STEMI patients in the rfsrc model. Incorporating a single patient into the model can yield a local explanation of each patient, thus guiding clinicians in developing precision treatments.

**Conclusion:**

The rfsrc model outperformed the coxph model in terms of predictive performance. Clinicians can use these predictive models to understand the major risk factors for each STEMI patient and thus develop more individualised and precise treatment strategies.

## 1 Introduction

ST-segment elevation myocardial infarction (STEMI), which is a deadly cardiovascular emergency, is often caused by thrombotic blockage of a coronary artery and necessitates prompt diagnosis and reperfusion treatment. STEMI survival rates have increased over the past few decades as a result of initial percutaneous coronary intervention (PCI) programs, contemporary antithrombotic treatment, and secondary preventive strategies (1, 2). Nonetheless, coronary artery disease continues to be the leading cause of death worldwide and significantly affects public health, mostly early mortality (1). Therefore, thinking about how to increase the survival rate and improve the prognosis of STEMI patients worthwhile.

The use of survival prediction models can help address this issue. Most survival prediction models are constructed primarily on the basis of traditional statistical methods. These models are further limited by the small number of variables for which they can account due to concerns about overfitting and multicollinearity, and these models require the statistical assumption of independent, linear connections between dependent and independent variables (3). Without being constrained by predetermined assumptions about data behaviour and variable preselection, machine learning (ML) algorithms build models by identifying or discovering underlying patterns in the data. Thus, ML is a potential solution for overcoming these limitations (4).

Predictive variables need to be screened before models are constructed. Traditional statistical techniques, such as univariate and multivariate regression analyses, are most often used in variable selection. Contradictory hazard ratios between univariate and multivariate Cox regressions are occasionally produced by these techniques. The multicollinearity between variables causes this contradiction, which skews the results (5). A regression-based approach that allows for the inclusion of a high number of variables in the model, the least absolute shrinkage and selection operator (LASSO), overcomes overfitting by creating a penalty function (6). Additionally, LASSO addresses multicollinearity problems, producing more pertinent predictive variables and compensating for the drawbacks of conventional techniques (7). In this study, LASSO regression was used to screen variables for the survival prediction models.

After the variables are screened, the method that will be used to build the predictive models is selected. In medicine, ML techniques are becoming recognised as useful instruments. These techniques enable the proper analysis of large datasets and promote the use of individualised and accurate medical approaches. However, traditional ML models lack interpretability, which makes it difficult for medical professionals to trust the models’ outcomes in diagnostic and decision-making (8). In compliance with the General Data Protection Regulation (GDPR), the European Union has established basic requirements for the use of ML systems in public health. One of these requirements is that the model must be explainable. In the field of artificial intelligence, explainable machine learning (XAI) is emerging as a potential study area. The goal of this area of research is to look for ways to analyse or supplement ML black box models so that the internal workings and results of algorithms may be made more understandable and visible (9). Recently, we developed an XAI package—the survex package—that improves the interpretability and transparency of predictive models and can be better applied in clinical work. To the best of our knowledge, the survex package has been applied in clear cell renal cell carcinoma, uveal melanoma, bone marrow transplantation, heart failure, etc., but it has not yet been applied in the field of STEMI (10–13). Therefore, two survival models, the Cox proportional hazards regression (coxph) model and the random survival forest (rfsrc) model, were constructed in this study using the variables screened by LASSO regression. These two survival models will be interpreted and compared with the survex package to help clinicians estimate the overall survival (OS) of STEMI patients as well as the determinants of OS.

## 2 Materials and methods

### 2.1 Study population

This was a retrospective study. We included 1,341 STEMI patients who underwent emergency coronary angiography and PCI at the First Affiliated Hospital of Kunming Medical University between June 2018 and January 2023. After admission, all patients received standardised treatment according to the recommended guidelines for STEMI. The inclusion criteria for this study were as follows: (i) the diagnosis of STEMI needed to meet the criteria of the 2023 ESC Guidelines for Management of Acute Coronary Syndromes (14); (ii) emergency PCI performed within 24 h of symptom onset. The exclusion criteria for this study were as follows: (i) loss to follow-up; (ii) missing essential data; and (iii) other serious comorbidities (e.g., severe hepatic and renal insufficiency, haematological disorders, malignant tumours, autoimmune diseases, and acute infections). In the end, the data from 893 STEMI patients who underwent emergency PCI were analysed in this study.

### 2.2 Data collection

A total of 144 variables, including data on demographic characteristics, history of other diseases, current treatment regimen, laboratory indicators, coronary angiography, electrocardiography, and echocardiography results, were collected from STEMI patients at the time of admission. After collating the data and removing variables with missing values, 56 variables remained. We made a baseline table of some of these 56 variables to help understand the general characteristics of the study population. These variables included age, sex, body mass index (BMI), blood pressure (BP), Killip class, medical history, red blood cells (RBCs), white blood cells (WBCs), neutrophils, lymphocytes, monocytes, haemoglobin, platelets, creatine kinase isoenzyme-MB (CKMB), myoglobin, troponin, and prothrombin time (PT); thrombin time (TT), activated partial thromboplastin time (APTT), alanine aminotransferase (ALT), aspartate aminotransferase (AST), albumin, globulin (GLB), conjugated bilirubin (CB), unconjugated bilirubin (UCB), uric acid, total cholesterol (TC), triglycerides (TG), high-density lipoprotein-C (HDL-C), low-density lipoprotein-C (LDL-C), estimated glomerular filtration rate (eGFR), number of stents implanted, Gensini Score, and global registry of acute coronary events (GRACE) Score.

All the blood samples were collected during an 8-to-12-h fasting period and were later dispatched to the laboratory of the First Affiliated Hospital of Kunming Medical University for additional analysis and testing. The investigators obtained survival data for this study by telephone follow-up with patients or their families, considering the patients who did not answer the phone as being lost to follow-up. Verbal informed consent was obtained from each patient by telephone, and all data were fully anonymised.

### 2.3 Outcome

OS was the study’s main outcome, and it was defined as the amount of time that passed between a STEMI patient’s discharge and their last follow-up visit or death from any cause.

### 2.4 Statistical methods

Comparison of the baseline characteristics of patients with STEMI: Results are displayed as means ± standard deviations for continuous variables with a normal distribution, and the t-test was used for intergroup comparisons. Continuous variables that did not follow a normal distribution are displayed as medians (P25, P75), and the Mann–Whitney U test was used to compare groups. Categorical variables are expressed as frequencies and percentages, and their intergroup comparisons were made using the Chi-square test.

Screening of variables for inclusion in the models: To choose variables associated with the OS of STEMI patients, contemporary statistical shrinkage techniques—especially LASSO regression—were used in the creation of the prediction models. LASSO regression analysis can be used for shrinkage and variable selection in linear regression models. By constraining the model parameters so that the regression coefficients for some variables decrease towards zero, LASSO regression analysis minimises the prediction error for a quantitative response variable, yielding a subset of predictors. Following the shrinkage process, variables with a regression coefficient of zero are removed from the model, whereas variables with a regression coefficient of nonzero have the strongest correlation with the response variable. The R software’s LASSO regression analysis selects the optimal lambda value after ten iterations of K cross-validation for the centralisation and normalisation of the included variables on the basis of the type measure of −2 log-likelihood and binomial family. “Lambda.lse” can provide a model with the fewest independent variables and high performance (15). Ultimately, we identified the most predictive variables on the basis of one standard error criterion.

Comparison and interpretation of the models: We constructed coxph and rfsrc models using variables screened by LASSO regression. First, the C-index, C/D AUC, and Brier score were used to evaluate the performance of the coxph and rfsrc models. Significance of performance differences was assessed via hypothesis testing: bootstrap test for C-index and C/D AUC (*α* = 0.05, 1,000 resamples) and Wilcoxon signed rank test for Brier score. Second, we utilised the partial dependence survival profile and the time-dependent variable importance to provide a global explanation for the whole cohort. Finally, a local explanation for a single patient was obtained with the SurvSHAP(t) and SurvLIME plots, together with the ceteris paribus survival profile. The X-axis in each graph shows the interval between discharge and the last follow-up visit or any cause of death. All event times are presented in red, whereas census times are presented in grey. IBM SPSS and Statistics version 26.0, R 4.3.2, was used to perform the statistical analysis in this study. A *p* value < 0.05 was considered to indicate a statistically significant difference, and all the statistical tests were two-tailed.

## 3 Results

### 3.1 Patient characteristics

After patients whose data were incomplete or who were lost to follow-up were excluded, this study ultimately included 893 patients with acute STEMI. Of these, 82 patients died, with a median OS of 8.5 months, and 811 patients survived, with a median OS of 37 months. Among the total number of patients, 755 (84.5%) were male, and 138 (16.5%) were female. The mean age was 60.57 ± 12.02 years. We divided the patients into a deceased group and a survivor group. Compared with the survivor group, the deceased group had lower RBC, haemoglobin, and albumin levels and higher monocyte, myoglobin, uric acid, and Gensini and GRACE Scores (*p* < 0.05). Additional demographic and clinical characteristics of the patients are shown in Table 1.

**Table 1:** Baseline characteristics.

Variables	Total (*n* = 893)	Deceased group (*n* = 82)	Survivor group (*n* = 811)	*p*
Basic characteristics				
OS (month)	35.00 (22.00, 51.00)	8.50 (0.00, 27.00)	37.00 (24.00, 52.00)	<0.001
Age (year)	60.57 ± 12.02	69.59 ± 11.21	59.66 ± 11.73	<0.001
Male	755 (84.5%)	58 (70.7%)	697 (85.9%)	<0.001
BMI (kg/m2)	24.32 ± 3.17	24.01 ± 2.88	24.35 ± 3.20	0.35
Systolic BP (mmHg)	126.47 ± 24.02	126.88 ± 23.16	126.43 ± 24.11	0.872
Diastolic BP (mmHg)	81.13 ± 16.28	78.87 ± 16.71	81.36 ± 16.23	0.186
Killip class				<0.001
I	589 (66.0%)	32 (39.0%)	557 (68.7%)	
II	215 (24.1%)	28 (34.1%)	187 (23.1%)	
III	43 (4.8%)	9 (11.0%)	34 (4.2%)	
IV	46 (5.2%)	13 (15.9%)	33 (4.1%)	
Medical history				
Heart failure	251 (28.1%)	45 (54.9%)	206 (25.4%)	<0.001
Hypertension	489 (54.8%)	56 (68.3%)	433 (53.4%)	0.010
Diabetes	278 (31.1%)	29 (35.4%)	249 (30.7%)	0.383
Hyperlipidemia	304 (34.0%)	13 (15.9%)	291 (35.9%)	<0.001
Stroke	43 (4.8%)	11 (13.4%)	32 (3.9%)	<0.001
Smoking	520 (58.2%)	36 (43.9%)	484 (59.7%)	0.006
Laboratory indicators				
RBC (10^12/L)	4.91 ± 0.70	4.69 ± 0.77	4.93 ± 0.69	0.002
WBC (10^9/L)	10.74 (8.27, 12.98)	10.81 (8.90, 14.32)	10.72 (8.23, 12.90)	0.225
Neutrophils (10^9/L)	8.26 (5.85, 10.57)	8.42 (6.04, 11.99)	8.26 (5.82, 10.42)	0.307
Lymphocytes (10^9/L)	1.57 (1.12, 2.04)	1.52 (1.08, 1.94)	1.58 (1.15, 2.05)	0.485
Monocytes (10^9/L)	0.56 (0.41, 0.80)	0.73 (0.48, 0.95)	0.56 (0.40, 0.79)	<0.001
Haemoglobin (g/L)	153.03 ± 23.99	144.56 ± 26.03	153.89 ± 23.62	0.001
Platelet (10^9/L)	217.00 (175.00, 267.00)	221.50 (180.75, 270.25)	217.00 (175.00, 267.00)	0.698
CKMB (ng/mL)	20.42 (3.56, 75.99)	15.79 (6.24, 69.51)	21.43 (3.36, 76.67)	0.716
Myoglobin (ng/mL)	154.00 (56.65, 360.45)	221.50 (108.62, 397.75)	140.00 (53.07, 355.00)	0.005
Troponin (ng/mL)	2.36 (0.12, 15.14)	3.80 (0.30, 16.55)	2.23 (0.10, 15.10)	0.323
PT (second)	13.78 ± 2.63	14.56 ± 3.81	13.70 ± 2.47	0.005
TT (second)	18.50 (17.30, 20.30)	18.45 (17.00, 19.82)	18.50 (17.30, 20.50)	0.355
APTT (second)	38.90 (35.40, 44.50)	40.85 (36.78, 45.38)	38.60 (35.30, 44.40)	0.048
ALT (IU/L)	42.10 (29.00, 63.00)	39.55 (27.75, 68.00)	43.00 (30.00, 62.40)	0.669
AST (IU/L)	70.00 (31.00, 169.60)	66.50 (36.75, 195.75)	70.00 (31.00, 167.70)	0.570
Albumin (g/L)	39.57 ± 4.96	37.87 ± 5.61	39.75 ± 4.87	0.001
GLB (g/L)	32.34 ± 5.70	33.52 ± 5.68	32.22 ± 5.70	0.049
CB (umol/L)	3.50 (2.40, 4.95)	3.75 (2.90, 5.38)	3.40 (2.40, 4.90)	0.053
UCB (umol/L)	8.00 (5.70, 12.10)	8.55 (6.15, 13.40)	8.00 (5.60, 11.90)	0.179
Uric acid (μmol/L)	387.60 (317.80, 471.60)	450.45 (345.58, 527.30)	382.00 (314.60, 462.20)	<0.001
TC (mmol/L)	4.50 ± 1.17	4.22 ± 1.07	4.52 ± 1.18	0.011
TG (mmol/L)	1.50 (1.06, 2.06)	1.36 (0.99, 1.84)	1.51 (1.07, 2.12)	0.034
HDL-C (mmol/L)	1.03 (0.88, 1.22)	1.02 (0.88, 1.19)	1.03 (0.88, 1.22)	0.652
LDL-C (mmol/L)	2.80 (2.17, 3.51)	2.48 (1.93, 3.29)	2.81 (2.21, 3.54)	0.026
eGFR (ml/min)	71.88 (55.20, 89.91)	50.37 (36.01, 72.10)	73.97 (57.28, 91.83)	<0.001
Coronary angiography data				
Number of stents Implanted	1.00 (1.00, 2.00)	1.00 (1.00, 2.00)	1.00 (1.00, 2.00)	0.292
Gensini Score	66.00 (42.00, 89.00)	81.00 (45.75, 104.00)	64.00 (42.00, 88.00)	0.011
GRACE Score	148.00 (129.00, 170.00)	182.00 (150.00, 207.00)	146.00 (127.00, 167.00)	<0.001

(1) If the data for continuous variables were normally distributed, independent samples t tests were employed; if not, Mann–Whitney U tests were utilised. Chi-square tests were used to analyze the differences between groups in categorical variables. *p* values were obtained by comparing the survivor group and the deceased group; p values less than 0.05 were considered statistically significant. (2) OS, overall survival; BMI, body mass index; BP, blood pressure; RBC, red blood cell count; WBC, white blood cell count; CKMB, creatine kinase isoenzyme-MB; PT, prothrombin timel; TT, thrombin time; APTT, activated partial thromboplastin time; ALT, alanine aminotransferase; AST, aspartate aminotransferase; GLB, globulin; CB, conjugated bilirubin; UCB, unconjugated bilirubin; TC, total cholesterol; TG, triglycerides; HDL-C, high density lipoprotein-C; LDL-C, low density lipoprotein-C; eGFR, estimated glomerular filtration rate.

### 3.2 Predictive indicators selected from LASSO regression

**Figure 1:**
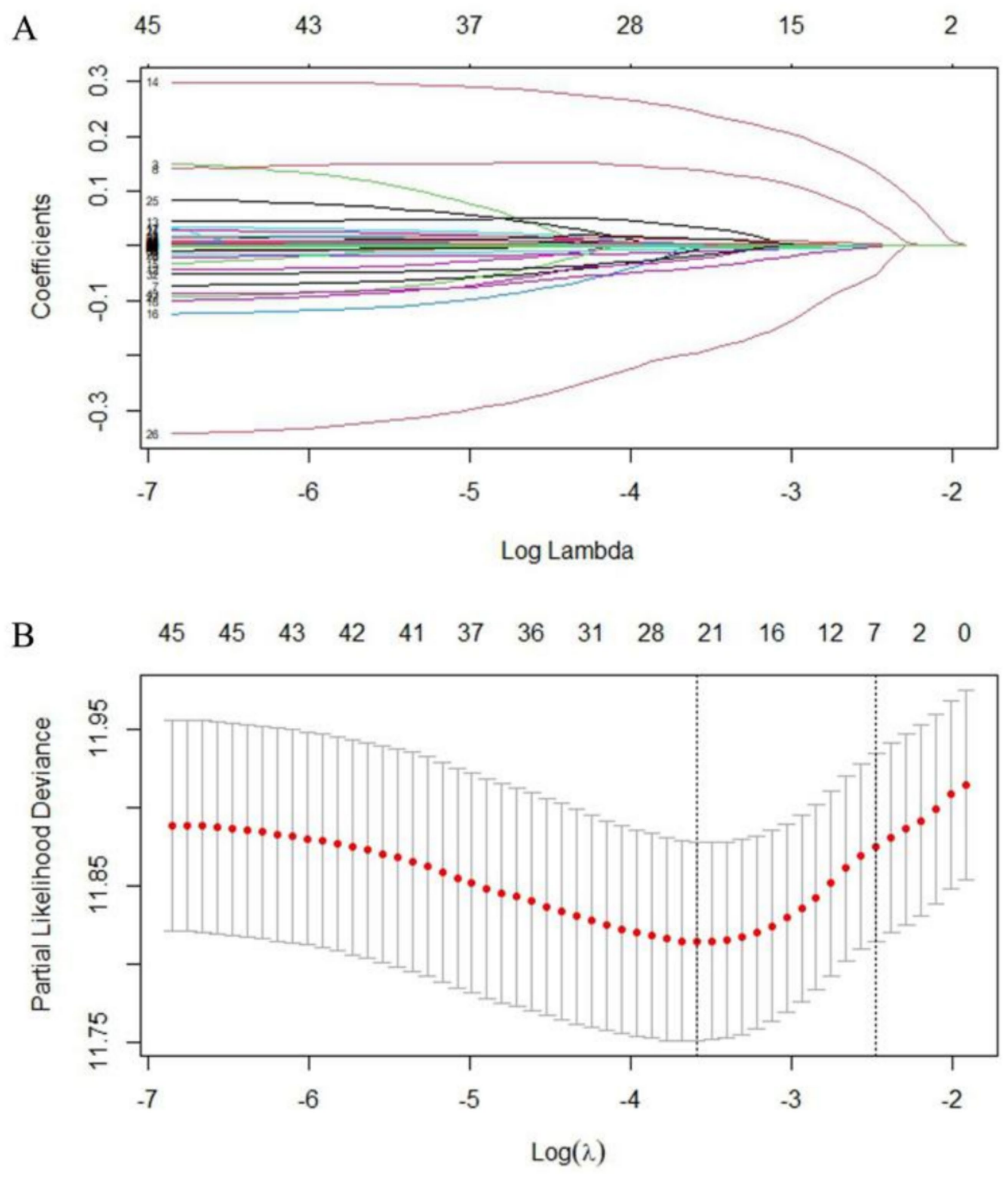
Screening of variables based on LASSO regression. **(A)** The variation characteristics of the coefficient of variables; **(B)** the selection process of the optimum value of the parameter *λ* in the LASSO regression model by cross-validation method.

In this study, we applied LASSO regression to screen the variables. Figure 1A, shows the variation characteristics of the coefficients of these variables in detail. Figure 1B displays the results of iterative analyses using the 10-fold cross-validation method, which identified 26 variables when the model error was minimal and 11 variables when the model error was one standard error. To make clinical application easier, the variables screened when log(*λ*) was one standard error, namely, diastolic blood pressure (DBP), Killip class, hyperlipidaemia, GRACE Score, CKMB, myoglobin, WBC, monocytes, TT, GLB, and CB, were ultimately selected.

### 3.3 Model performance for the whole cohort

**Figure 2:**
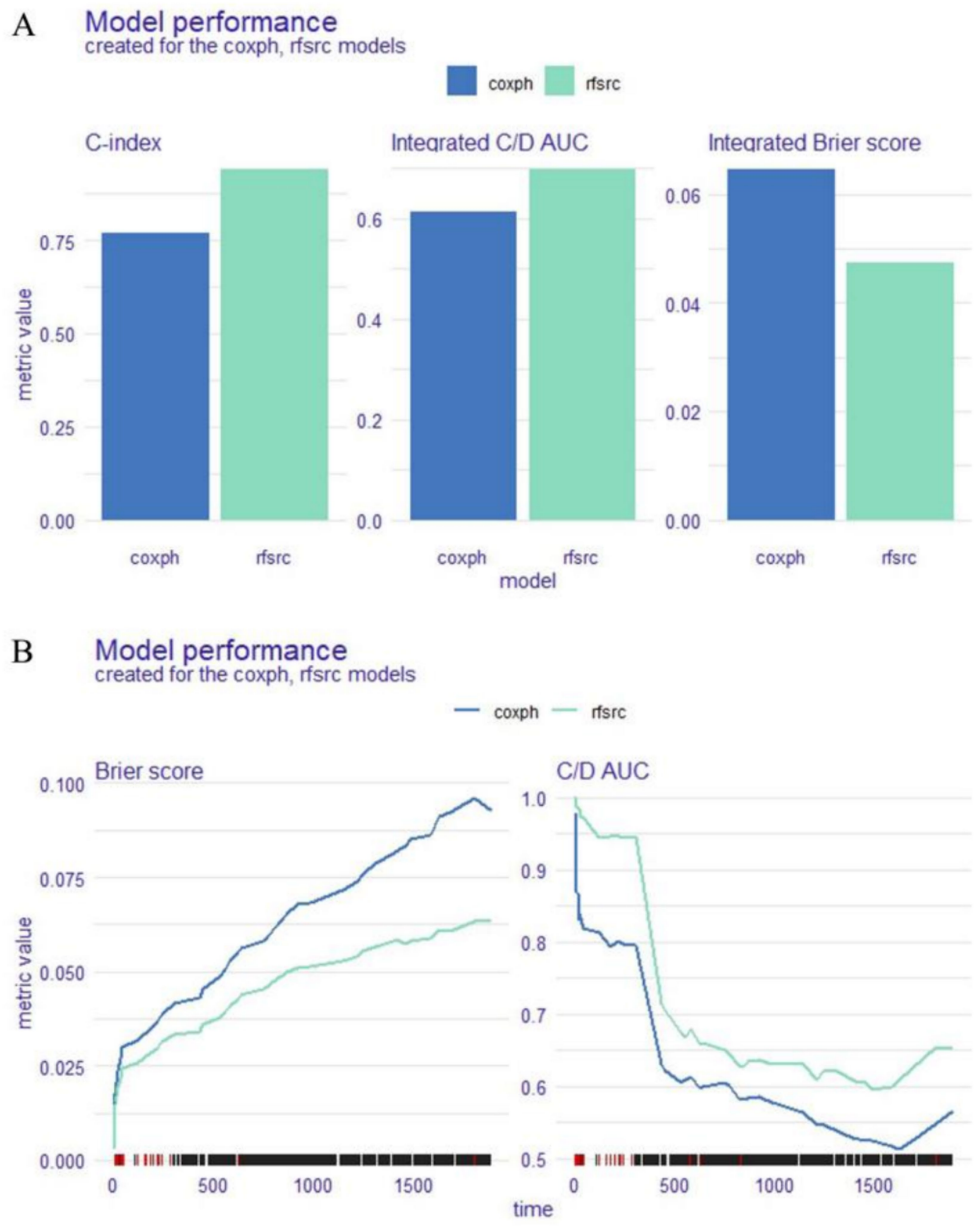
Model performance for the whole cohort. Explainable machine learning (XAI) data are shown as bar plots **(A)** and a time-dependent estimation **(B)**.

We used 11 variables selected by LASSO regression (DBP, Killip class, hyperlipidaemia, GRACE Score, CKMB, myoglobin, WBC, monocytes, TT, GLB, and CB) to construct two survival models (coxph and rfsrc) to predict the survival and prognosis of STEMI patients. Next, we utilised three methods, namely, the C-index, Brier score, and C/D AUC, to estimate the performance of the two models. The lower the Brier score was, the better the model performance was, and the higher the C/D AUC and C-index values were, the better the model performance was. The C-index was 0.771, the C/D AUC was 0.613, and the Brier score was 0.063 for coxph. The C-index was 0.941, the C/D AUC was 0.698, and the Brier score was 0.047 for rfsrc. We assessed the significance of the difference in performance between the two models (using 1,000 repetitions of the paired Bootstrap test for the C index and the C/D AUC and the Wilcoxon signed rank test for the Brier scores), and the results, as shown in Table 2, indicate that the rfsrc demonstrated statistically superior performance over coxph (<0.05). Combining the above findings, we can conclude that the model performance of rfsrc is better than the coxph for every measure (Figure 2A) and the duration of follow-up (Figure 2B).

**Table 2:** Comparison of performance metrics of coxph and rfsrc models.

Metric	coxph vs rfsrc Difference (95% CI)	*p* value
C-index	−0.170 (−0.218, −0.129)	<0.001^*^
C/D AUC	−0.195 (−0.198, −0.193)	<0.05^*^
Brier score	0.0082 (0.0044, 0.0124)	0.009^#^

^*^p values were derived from comparisons using the paired bootstrap test (1,000 iterations).

^#^*p* values were derived from comparisons using the Wilcoxon signed rank test.

CI, confidence interval.

### 3.4 Global explanation: time-dependent feature importance for the whole cohort

**Figure 3:**
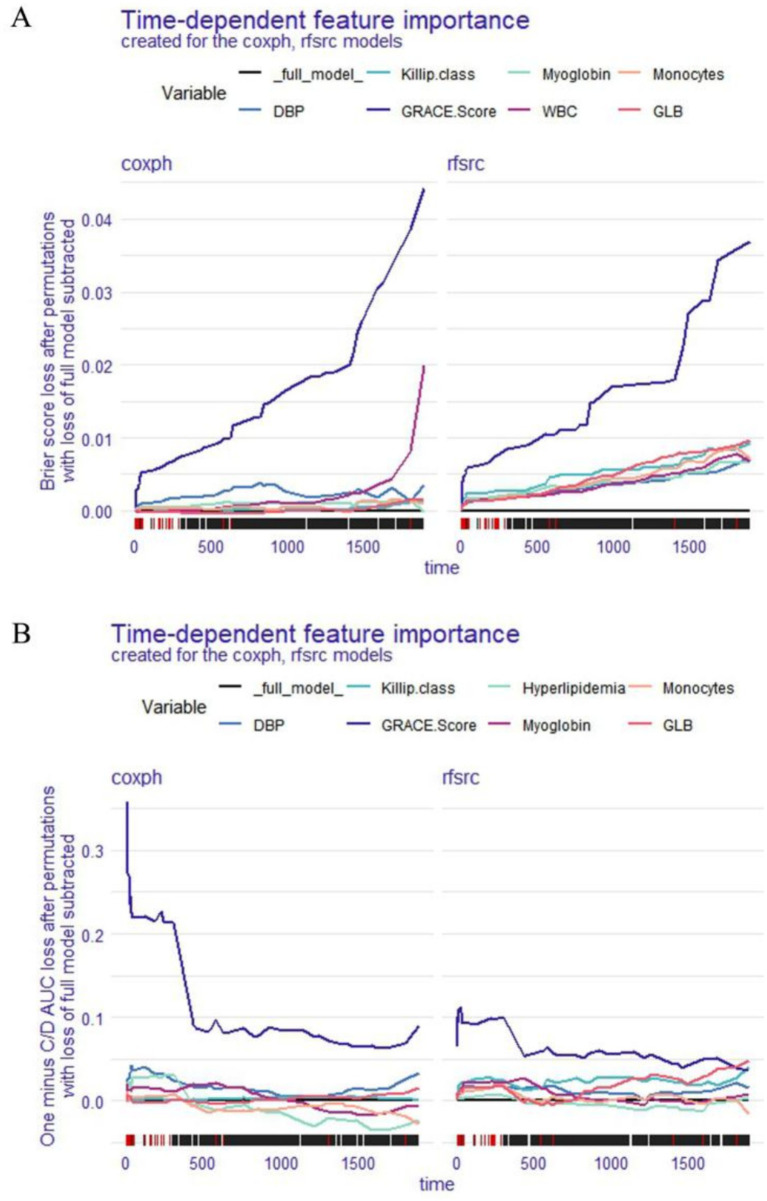
Global explanation: time-dependent feature importance and for the whole cohort, Brier score loss after permutation **(A)** and C/D AUC loss after permutation **(B)**.

Two techniques were used to evaluate the significance of the time-dependent variables for the entire cohort: Brier score loss after permutation and C/D AUC loss after permutation. The loss function’s change after each covariate’s replacement is shown on the y-axis. Variable significance is subject to variation over time; higher values of the loss function suggest that the variable has a greater impact on OS. The results of the Brier score loss after permutation (Figure 3A) and the C/D AUC loss after permutation (Figure 3B) revealed that, in both the coxph and the rfsrc models, the GRACE Score had the greatest effect on the OS of patients with STEMI.

### 3.5 Global explanation: partial dependence survival profile for the whole cohort

**Figure 4:**
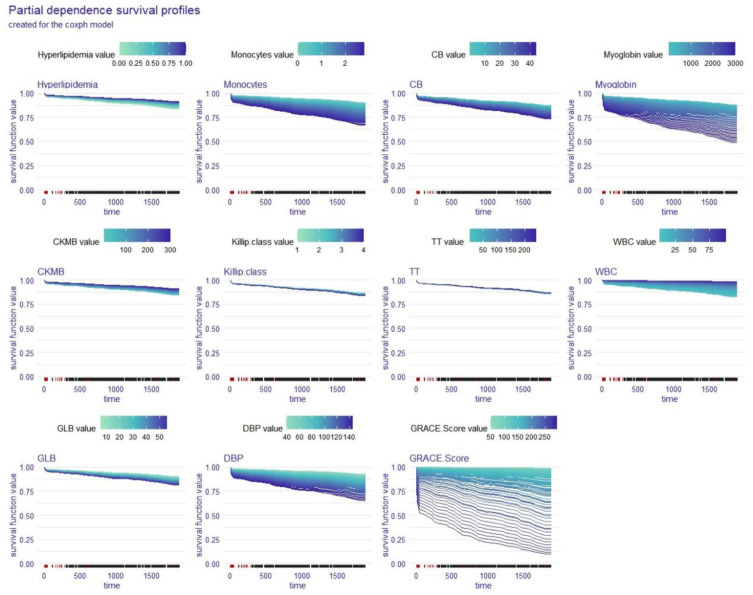
Global explanation: partial dependence survival profile for the whole cohort; coxph model. CB, conjugated bilirubin; CKMB, creatine kinase isoenzyme-MB; TT, thrombin time; WBC, white blood cell count; GLB, globulin; DBP, diastolic blood pressure.

**Figure 5:**
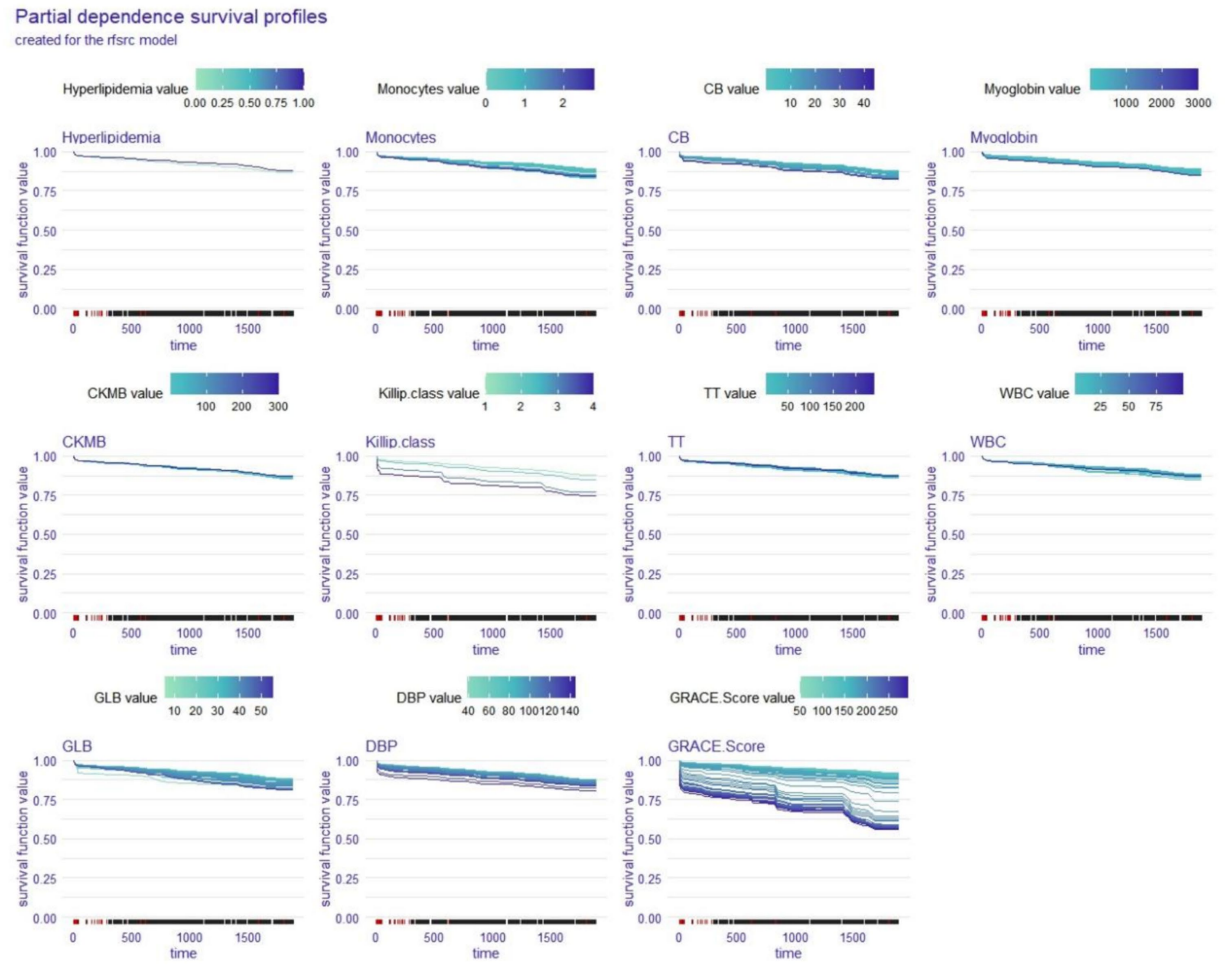
Global explanation: partial dependence survival profile for the whole cohort; rfsrc model. CB, conjugated bilirubin; CKMB, creatine kinase isoenzyme-MB; TT, thrombin time; WBC, white blood cell count; GLB, globulin; DBP, diastolic blood pressure.

The partial dependence survival profiles (PDPs) show how changes in one variable while all other factors remain the same affect the whole cohort’s OS. The larger the difference in a variable’s value was, the greater the impact that the variable had on OS, and the wider the region of the curve was. Figures 4, 5 show that DBP, GRACE Score, myoglobin, and monocytes had a significant effect on the OS of STEMI patients in the coxph model while DBP, GRACE Score, and GLB were the variables with a significant impact on the OS of STEMI patients in the rfsrc model. Among these factors, the GRACE Score has the widest curve area in both the coxph model and the rfsrc model, suggesting that it is the most important factor influencing the OS of STEMI patients.

### 3.6 Local explanation: SurvSHAP(t) plot for a single patient

**Figure 6:**
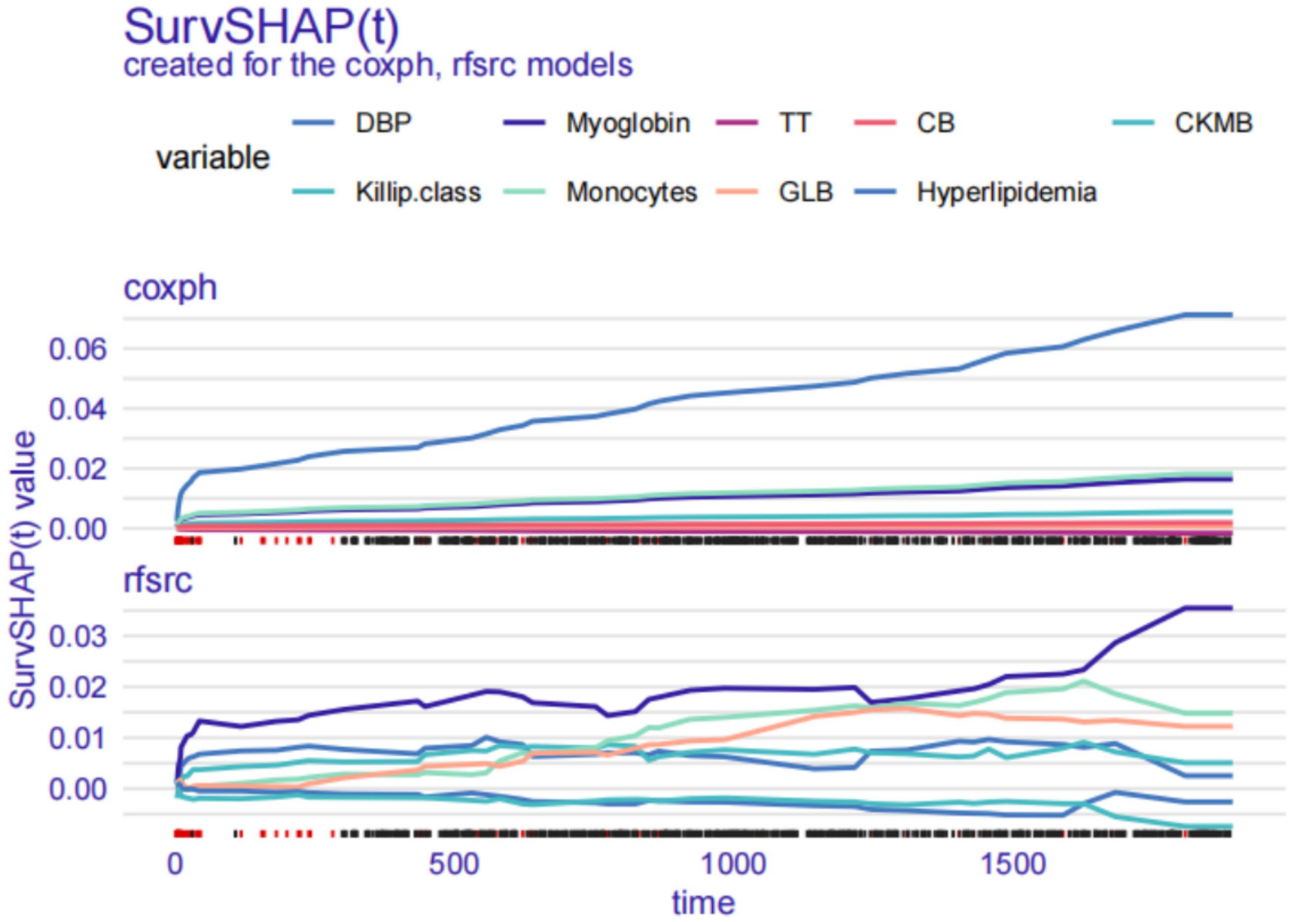
Local explanation: SurvSHAP(t) plot for a single patient. DBP, diastolic blood pressure; TT, thrombin time; CB, conjugated bilirubin; CKMB, creatine kinase isoenzyme-MB; GLB, globulin.

SurvSHAP(t) plots may be applied to analyse the relative contributions of each risk factor to OS across time for a particular patient. Every factor’s SurvSHAP(t) value is shown on the y-axis: a positive number suggests that the factor increased the patient’s OS, whereas a negative number suggests that the factor decreased the OS. The inclusion of STEMI Patient #204 (DBP 51 mmHg, Killip class I, GRACE Score 181, no hyperlipidaemia, CKMB 7.40 ng/mL, myoglobin 51.76 ng/mL, WBC 6.38 × 10^9/L, monocytes 0.40 × 10^9/L, TT 15.60 s, GLB 32.3 g/L, and CB 3.60 μmol/L) in the survival model enabled it to transition from predicting outcomes for the whole cohort to specific individuals. According to Patient #204’s SurvSHAP(t) plot, the absence of hyperlipidaemia increased the patient’s chances of survival in the coxph model, whereas myoglobin increased the patient’s chances of survival in the rfsrc model (Figure 6).

### 3.7 Local explanation: SurvLIME plot for a single patient

**Figure 7:**
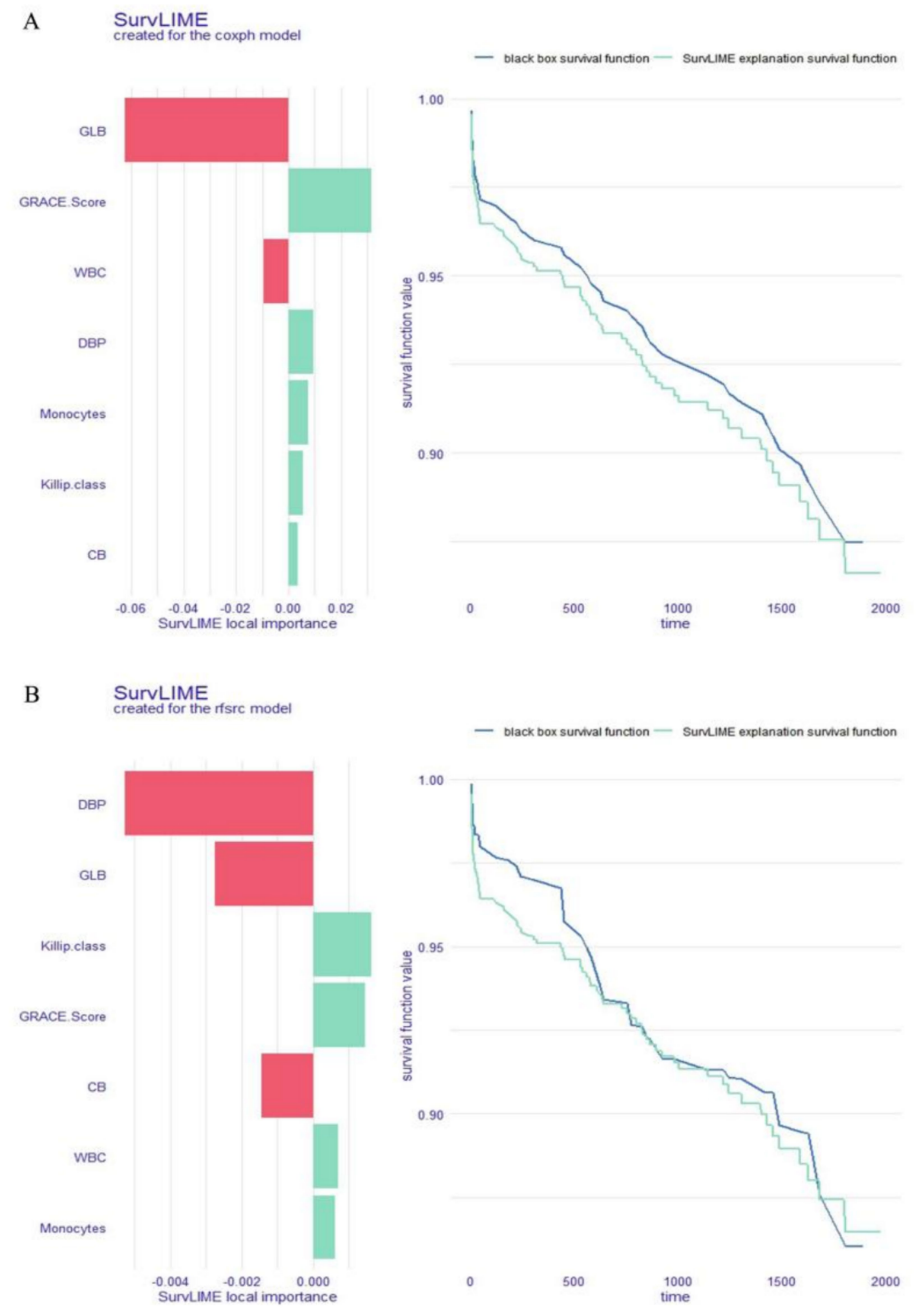
Local explanation: SurvLIME plot for a single patient; coxph model **(A)** and rfsrc model **(B)**. GLB, globulin; WBC, white blood cell count; DBP, diastolic blood pressure; CB, conjugated bilirubin.

In addition to the SurvSHAP(t) plot, the SurvLIME plot can also be used to identify the predictors that have the greatest effects on the OS of a particular patient. Each variable’s influence on a selected patient’s survival is shown on the SurvLIME plot’s left. A larger area indicates a greater impact on the patient’s OS and a higher SurvLIME local significance value indicates a worse chance of survival for the patient. The black-box model’s predictions and those of the coxph or rfsrc models are compared in the right section: the model’s outcomes are more precisely described when the two functions are closer. Following Patient #204 into the rfsrc and coxph models, two SurvLIME plots were produced (Figures 7A,B). Drawing conclusions from Figure 7A, we may infer that in the coxph model, the GRACE Score lowers the patient’s odds of survival, whereas GLB increases them. In the rfsrc model, Figure 7B shows that while DBP and GLB increase the patient’s odds of survival, the Killip class and GRACE Score decrease those odds. The estimate of patient survival may be considered relatively accurate because these two functions are somewhat close.

### 3.8 Local explanation: ceteris paribus survival profile for a single patient

**Figure 8:**
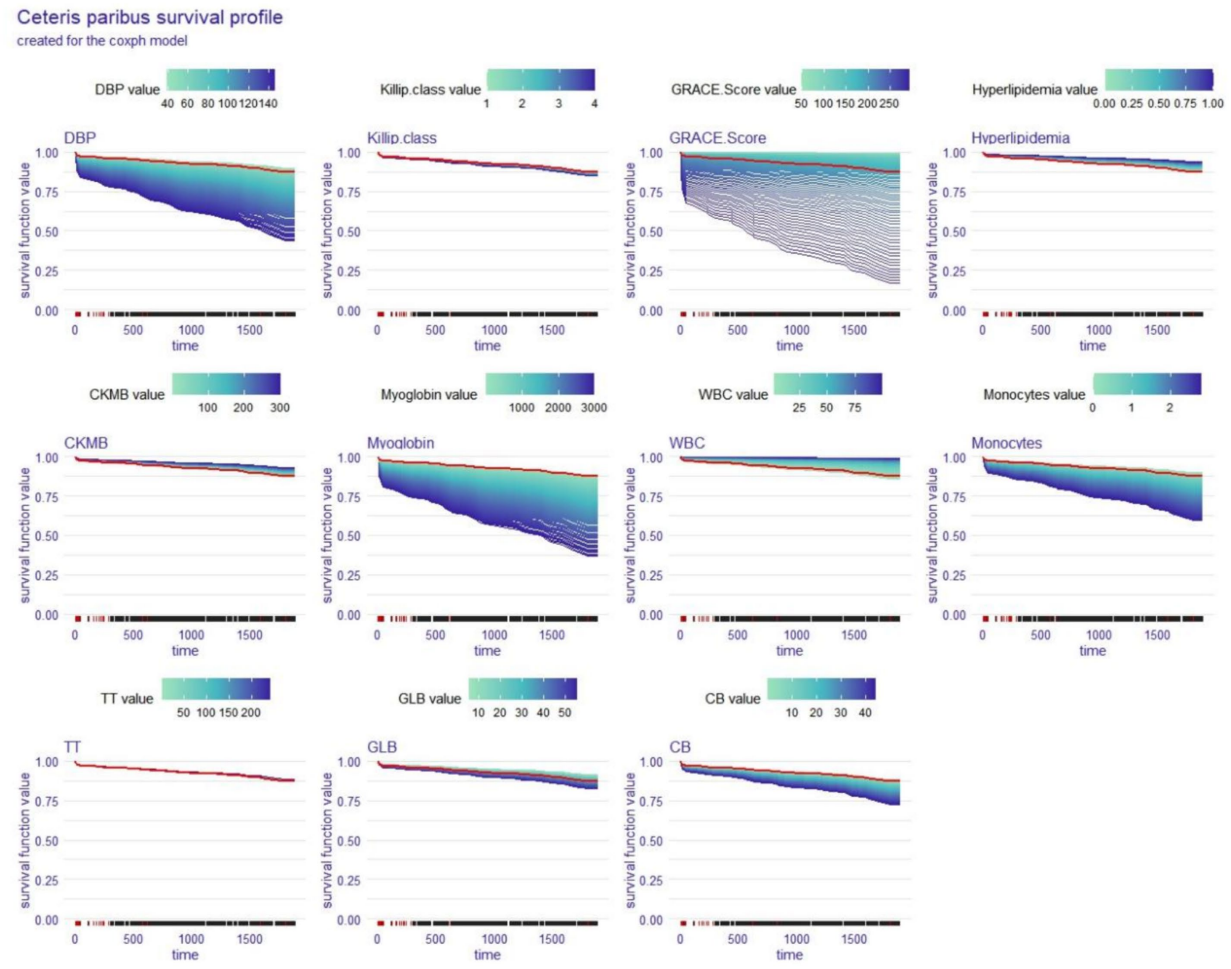
Local explanation: ceteris paribus survival profile for a single patient; coxph model. DBP, diastolic blood pressure; CKMB, creatine kinase isoenzyme-MB; WBC, white blood cell count; TT, thrombin time; GLB, globulin; CB, conjugated bilirubin.

**Figure 9:**
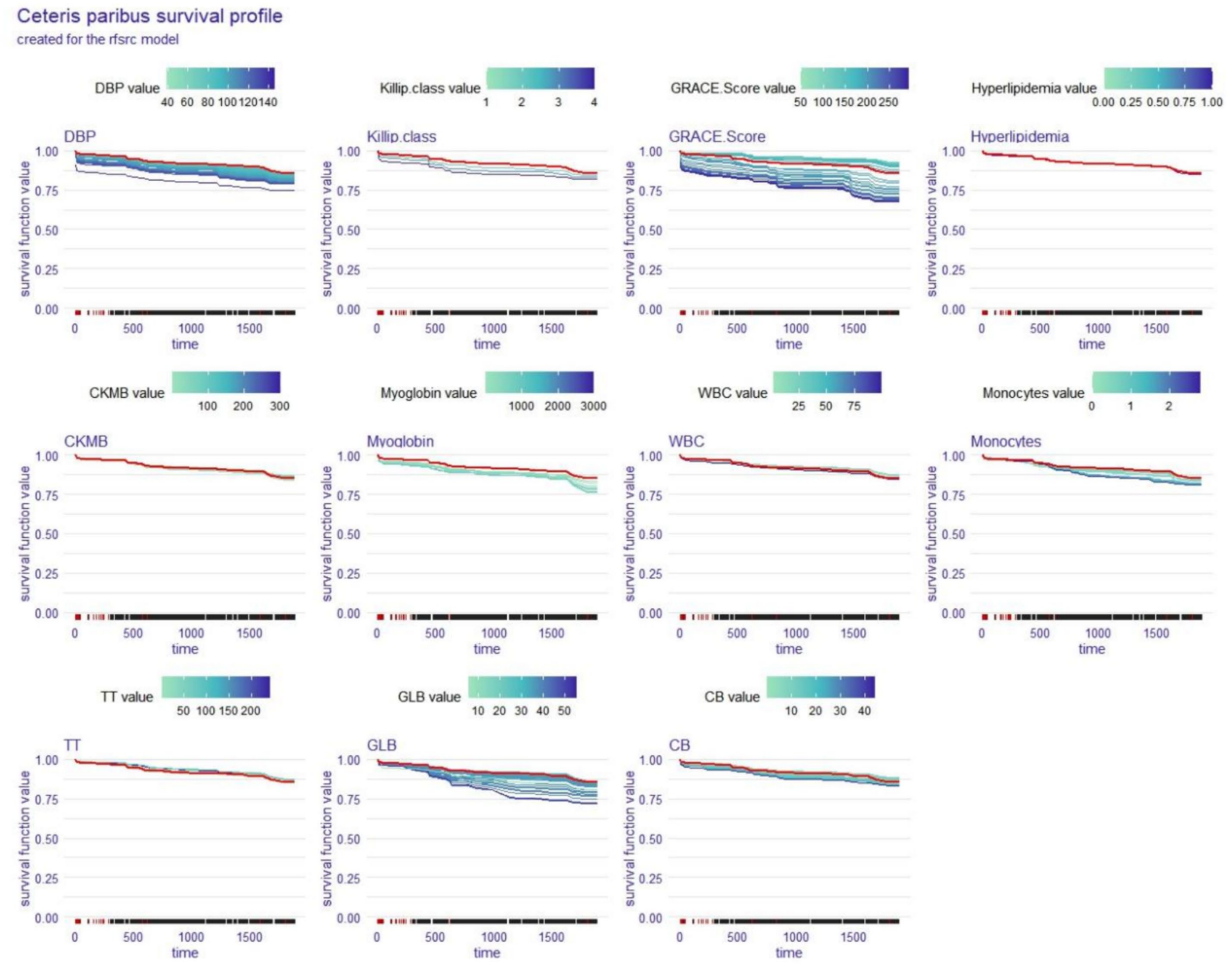
Local explanation: ceteris paribus survival profile for a single patient; rfsrc model. DBP, diastolic blood pressure; CKMB, creatine kinase isoenzyme-MB; WBC, white blood cell count; TT, thrombin time; GLB, globulin; CB, conjugated bilirubin.

The ceteris paribus survival profile (CPP) is a PDP equivalent that can only be used on a single subject at a time. Similar to PDP, patients’ OS decreased as the CPP function’s y-axis values decreased, and the variables that had the greatest interlevel variability also had the greatest effects on OS. We again analysed Patient #204 with the coxph and rfsrc models to obtain two CPPs (Figures 8, 9), and the red line indicates the value corresponding to this patient in each variable.

## 4 Discussion

Acute myocardial infarction is a major cause of morbidity and mortality worldwide (16, 17). The mortality rate after STEMI has decreased as a result of developments in early reperfusion therapy and adjunctive medication. Nevertheless, low- and middle-income countries have not seen comparable advances (18). Therefore, it is necessary to construct models using XAI techniques to predict the prognosis of STEMI patients. This helps clinicians determine the primary risk factors contributing to mortality, thus helping to identify high-risk groups that require enhanced treatment regimens and close follow-up.

In the present study, we screened the best predictor variables (DBP, Killip class, hyperlipidaemia, GRACE Score, CKMB, myoglobin, WBC, monocytes, TT, GLB, and CB) using LASSO regression. These variables were then used to construct two models, namely, the coxph and rfsrc models, to predict the OS of patients with STEMI. Finally, we utilised the survex package to compare the two survival prediction models and interpret the predicted results, which can help clinicians implement clinical decisions more accurately. The use of the survex package is divided into the following three sections.

In the first part, three criteria, namely, the C-index, C/D AUC and Brier score, were utilised to assess the performance of the coxph and rfsrc models. The results of this study show that the rfsrc model has higher C-index and C/D AUC values and lower Brier scores than the coxph model, and the difference in the performance of the two models is statistically significant, indicating that the rfsrc model performs better and is more predictive than the coxph model.

In the second part, a variety of global explanations of the coxph and rfsrc models were conducted to investigate the predictive power of the models for the whole patient population. We utilised two different loss functions (the Brier score and the 1-CD/AUC) to assess the significance of each variable in the models, which involves a process of change over time. According to the Brier score loss and the C/D AUC loss after permutation, the GRACE Score had the greatest impact on the OS of STEMI patients in the both coxph and rfsrc models. Furthermore, the PDPs showed that DBP, GRACE Score, myoglobin, and monocytes had a significant effect on the OS of STEMI patients in the coxph model, while DBP, GRACE Score, and GLB were the variables with a significant effect on the OS of STEMI patients in the rfsrc model. Among these variable, the GRACE Score had the widest area of the curve in both models, reconfirming that the GRACE Score has the most important influence on the OS of STEMI patients. This finding reminds us that the GRACE Score is the first thing that should be considered when assessing the OS of STEMI patients. The GRACE Score is calculated from eight variables, including age, cardiac arrest on admission, Killip class, ST-segment deviation, creatinine level, elevated cardiac enzymes, heart rate and systolic blood pressure. Several studies have shown that the GRACE Score is the best predictor of in-hospital death and 6-month postdischarge prognosis in patients with acute coronary syndrome (19, 20).

Consistent with Hung J et al.’s (21) multi-center validation, the GRACE score remained the strongest univariate predictor of OS in our LASSO-selected feature set. This reaffirms its irreplaceable role in STEMI risk stratification. Unlike previous studies (22, 23), while reaffirming the importance of the GRACE score, our LASSO regression identifies an additional set of variables that, in combination with the GRACE score, provide the best set of predictions for our model. In this study, these variables were applied to the XAI models to visualise risk factors, which not only helps clinicians to comprehensively assess patients from various aspects to identify early high-risk patients but also solves the problem of delayed risk assessment and the “actionability gap” pointed out by the 2023 ESC guideline (14), and achieves precise interventions targeting patient-specific risk factors.

In the third part, we use several local explanation techniques to better understand how the model predicts a particular patient’s circumstances. The SurvSHAP(t) function is used to analyse the effect of each risk factor on OS for a specific patient at different time points. The SurvLIME function is able to reveal the significance of each risk factor in the OS of selected patients and the positive or negative impact of changes in these factors over time on the predicted result. Similar to the PDP for the entire cohort, the CPP makes it possible to visually quantify the contribution of each risk factor to OS for each selected patient.

Through the above series of operations, we can accurately determine the main factors that affect the OS of STEMI patients and the extent to which these factors affect OS. This approach can even identify important factors that affect the OS of individual patients, for whom clinicians can develop individualised treatment plans, thus enabling precision medicine to help improve patient prognosis and survival.

However, there are several limitations to this study. First, this study is a retrospective observational study, which is inevitably subject to a certain degree of bias. In the future, we can perform a prospective study to validate the two prediction models. Second, this was a single-centre study. Although the ML model showed outstanding predictive ability, there is a need for future validation using multicentre datasets to further refine the predictive models. Third, critical pre-hospital time intervals (pain-to-first-medical-contact time and pain-to-PCI time) were not available in our dataset, preventing assessment of their impact on outcomes. Finally, our study did not capture STEMI network-level variables (e.g., direct admission vs. transfer status, hub-spoke designation), limiting analysis of system-level efficiencies. Future prospective studies should prioritise collecting these metrics to validate our model across care pathways.

## 5 Conclusion

In this study, we used LASSO regression to screen 11 predictor variables to construct the models. By comparison, the rfsrc model was comprehensively superior to the coxph model. We then performed global and local explainability analyses of the predictive models using the survex package. As shown by the analysis, our models can provide valuable predictive information not only for the entire STEMI patient population but also for a single specific STEMI patient, thus providing important guidance for clinicians in developing individualised and precise treatment plans.

## Data Availability

The raw data supporting the conclusions of this article will be made available by the authors, without undue reservation.

## Glossary

STEMI: Acute ST-segment elevation myocardial infarction
OS: Overall survival
PCI: Percutaneous coronary intervention
LASSO: Least absolute shrinkage and selection operator
coxph: Cox proportional hazards regression
rfsrc: Random survival forest
DBP: Diastolic blood pressure
GRACE: Global registry of acute coronary events
GLB: Globulin
ML: Machine learning
GDPR: General Data Protection Regulation
XAI: Explainable machine learning
BMI: Body mass index
BP: Blood pressure
RBCs: Red blood cells
WBCs: White blood cells
CKMB: Ccreatine kinase isoenzyme-MB
PT: Prothrombin time
TT: Thrombin time
APTT: Activated partial thromboplastin time
ALT: Alanine aminotransferase
AST: Aspartate aminotransferase
CB: Conjugated bilirubin
UCB: Unconjugated bilirubin
TC: Total cholesterol
TG: Triglycerides
HDL-C: High-density lipoprotein-C
LDL-C: Low-density lipoprotein-C
eGFR: Estimated glomerular filtration rate
PDP: Partial dependence survival profile
CPP: Ceteris paribus survival profile
